# Screening of Lipid Metabolism-Related Gene Diagnostic Signature for Patients With Dilated Cardiomyopathy

**DOI:** 10.3389/fcvm.2022.853468

**Published:** 2022-04-01

**Authors:** Man Xu, Ying-ying Guo, Dan Li, Xian-feng Cen, Hong-liang Qiu, Yu-lan Ma, Si-hui Huang, Qi-zhu Tang

**Affiliations:** ^1^Department of Cardiology, Renmin Hospital of Wuhan University, Wuhan, China; ^2^Hubei Key Laboratory of Metabolic and Chronic Diseases, Wuhan, China

**Keywords:** dilated cardiomyopathy, GEO, lipid metabolism, diagnosis, bioinformatics

## Abstract

**Background:**

Dilated cardiomyopathy (DCM) is characterized by enlarged ventricular dimensions and systolic dysfunction and poor prognosis. Myocardial lipid metabolism appears abnormal in DCM. However, the mechanism of lipid metabolism disorders in DCM remains unclear.

**Methods:**

A gene set variation analysis (GSVA) were performed to estimate pathway activity related to DCM progression. Three datasets and clinical data downloaded from the Gene Expression Omnibus (GEO), including dilated cardiomyopathy and donor hearts, were integrated to obtain gene expression profiles and identify differentially expressed genes related to lipid metabolism. GO enrichment analyses of differentially expressed lipid metabolism-related genes (DELs) were performed. The clinical information used in this study were obtained from GSE21610 dataset. Data from the EGAS00001003263 were used for external validation and our hospital samples were also tested the expression levels of these genes through RT-PCR. Subsequently, logistic regression model with the LASSO method for DCM prediction was established basing on the 7 DELs.

**Results:**

GSVA analysis showed that the fatty acid metabolism was closely related to DCM progression. The integrated dataset identified 19 DELs, including 8 up-regulated and 11 down-regulated genes. A total of 7 DELs were identified by further external validation of the data from the EGAS00001003263 and verified by RT-PCR. By using the LASSO model, 6 genes, including CYP2J2, FGF1, ETNPPL, PLIN2, LPCAT3, and DGKG, were identified to construct a logistic regression model. The area under curve (AUC) values over 0.8 suggested the good performance of the model.

**Conclusion:**

Integrated bioinformatic analysis of gene expression in DCM and the effective logistic regression model construct in our study may contribute to the early diagnosis and prevention of DCM in people with high risk of the disease.

## Introduction

Dilated cardiomyopathy (DCM) is one of the main indications for heart transplantation, which is characterized by ventricular enlargement and myocardial dysfunction (1). High incidence rate and mortality rate emphasize the need for heart transplantation in DCM.

In healthy adult hearts, fatty acids are the main energy substrates and β-oxidation provides 50–70% of the ATP required by the heart. It has been reported that in animal models of heart failure, the benefits of fatty acid utilization translate into more effective glucose metabolism due to decreased myocardial triglycerides and total fatty acid content (2). During the progression of heart failure, the transcription level of fatty acid oxidation is gradually down-regulated (3). ApoE abnormality is a well-known risk factor for cardiovascular disease, and rats lacking ApoE showed cardiac dysfunction after high-fat diet (4). Accumulating evidence shows that fatty acid oxidation disorders can worsen heart function and lead to decreased contractility (5, 6). Experimental and clinical data indicated that lipid accumulation leads to or worsens cardiac dysfunction, a process known as cardiac lipotoxicity (7). The level of partial lipid peroxides in the left ventricle of DCM and ICM patients was higher than that of the control group (6).

This was the first study to screen for abnormal expression of genes related to lipid metabolism in patients with DCM by combining bioinformatics and RT-PCR analyses, which indicated its diagnostic significance.

## Materials and Methods

### Human Heart Samples

Human heart samples were collected from the left ventricles of patients with DCM and healthy donors who had died unexpectedly and were unsuitable for transplantation for non-cardiac reasons. All procedures involving human samples were in accordance with the declaration of Helsinki, and informed consent for all donors’ hearts was obtained from their families. This study was approved by the review committee of the People’s Hospital of Wuhan University. The source of these donors has been previously described (8–10).

### Data Collection

Expression profiles for GSE79962, GSE21610, and GSE17800 were downloaded from the Gene Expression Omnibus (GEO) database. These three Expression profiles were then integrated to one expression profile containing 97 samples, including 70 DCM samples and 27 normal samples. In addition, the expression profile for EGAS00001003263 was obtained from European Genome-phenome Archive (EGA), which contains 65 end-stage DCM patients and 15 non-DCM controls (11). The “Limma” package in R software (version 3.5.1) was used to identify differentially expressed genes (DEGs) and the package “heatmap.2” was used to perform the hierarchical clustering analysis. Relative gene expression values with adjusted p-values < 0.05 and | log2 FC| > 0.5 (FC, fold change) were considered as DEGs.

After preprocessing, a total of 1206 lipid metabolism-related genes (DELs) were collected from the KEGG database and Molecular Signature Database (MSigDB version 7.4) (Supplementary Table 1) (12).

### Functional Annotation of the Differentially Expressed Genes

Gene Ontology (GO) analysis, including biological process, molecular function and cellular component were carried out using the clusterProfiler package of R language. A *p* < 0.05 was set as the cutoff criterion.

### RNA Extraction and RT-qPCR

A portion of the heart tissue was cut, added to Trizol reagent (Invitrogen, Carlsbad, United States) and then ground at low temperature to extract RNA. Thereafter, 1 μg of RNA was reverse-transcribed (RT) using the PrimeScript RT reagent kit with gDNA Eraser (Cat. No. RR047A; Takara Bio, Inc). Then ddH2O was added to the cDNA and diluted to 80 μl. The LightCycler 480 SYBR Green 1 Master Mix (04887352001, Roche) was used for qPCR. The following thermal cycling conditions were used: 95°C for 15 s, 55°C for 15 s, and 72°C for 15 s, for 40 cycles. Gene expression values were normalized to GAPDH values using the 2 - ΔΔCt method. Primer sequences for RT-qPCR are shown in Supplementary Table 2.

### Logistic Regression Model

**FIGURE 1:**
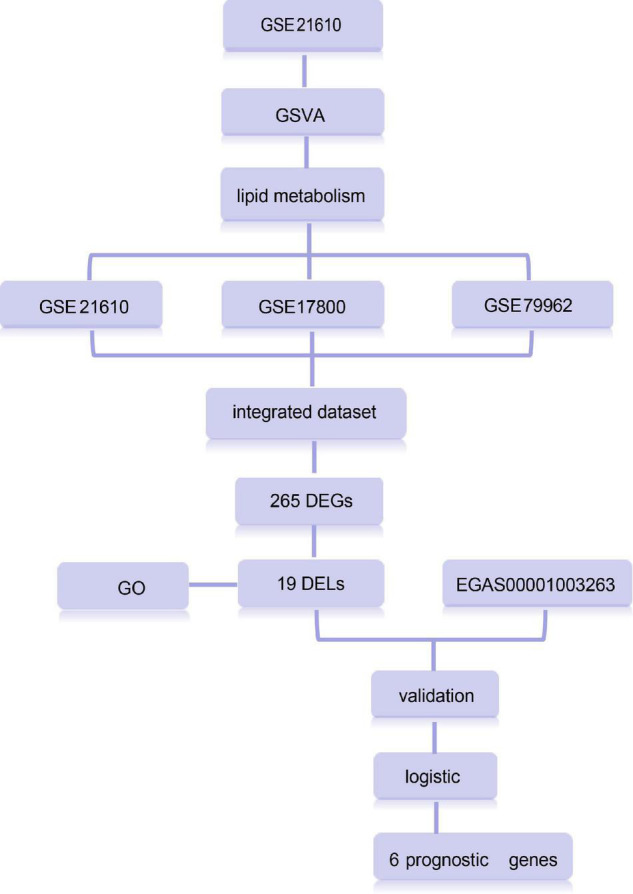
Overview of study design.

Dilated cardiomyopathy samples and healthy samples were considered as categorical responsive values and gene expression values as continuous predictive variables. The clinical characteristics of the samples are listed in Supplementary Table 3. Based on age and gender, the samples were randomly divided into the training and test set in a ratio of approximately 3:2 to construct and validate the model (Supplementary Table 4). The least absolute shrinkage and selection operator (LASSO) regression was carried out using the “glmnet” package in R to Calculate and select linear models and retain valuable variables. A binomial distribution variable was then used in the LASSO classification, as well as the 1 standard error of the minimum criteria (the 1-SE criteria) lambda value used to build the model with good performance but the least number of variables for 10-fold cross-validation. AUC values were used to evaluate the model’s ability to differentiate between DCM and normal samples by the pROC package in R (13, 14). The data analysis process is depicted in Figure 1.

### Statistical Analysis

Statistical analyses were performed using R software (version 3.6.2). Gene expression levels of the clinical samples were compared using Student’s *t*-test. Correlation between the expression of DELs was analyzed using Pearson’s correlation analysis. A Pearson’s correlation coefficient ranging between 0.5 and 1 indicated a strong correlation. A *p*-value < 0.05 was considered to be statistically significant.

## Results

### Differentially Expressed Lipid Metabolism-Related Genes of Dilated Cardiomyopathy

**FIGURE 2:**
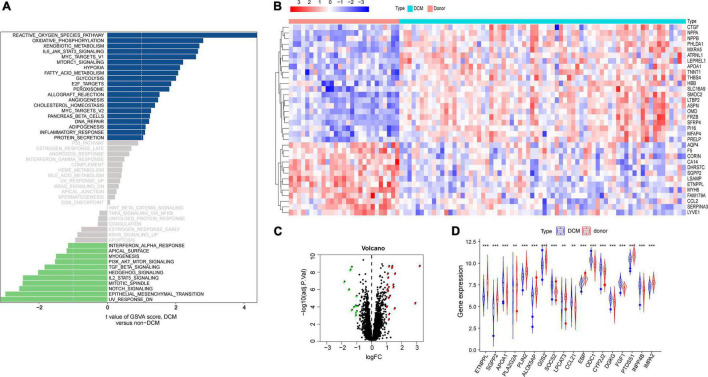
Identification of differentially expressed lipid metabolism-related genes (DELs) in DCM and healthy samples. **(A)** Histogram of the enrichment of Gene set variation analysis (GSVA). **(B)** Heatmap of the top 40 differentially expressed genes (DGEs) in the integrated microarray dataset. **(C)** Volcano plot of the 222 DGEs. The red dots represent the significantly up-regulated genes and the blue dots indicate the significantly down-regulated genes. **(D)** The boxplot of 18 differentially expressed lipid metabolism-related genes in the integrated microarray dataset.

To evaluate the potential mechanisms contributing to DCM, the GSE79962 gene set was applied to pathway analyses by using the GSVA R package. The results of GSVA analysis showed fatty acid metabolism as the relatively significant signature in DCM (Figure 2A), which suggested that fatty acid metabolism may contribute to DCM.

To further investigate lipid metabolism signaling pathways in DCM, three microarray datasets (GSE79962, GSE21610, and GSE17800) were integrated to obtain differentially expressed genes (DEGs). Heatmap (Figure 2B) and volcano (Figure 2C) were plotted based on the following criteria: | logFC | > 0.5 and an adjusted *p*-value < 0.05. A total of 265 DEGs were identified in the integrated dataset, of which 19 were differentially expressed lipid metabolism-related genes (DELs). The box plots displayed expression patterns of eight up-regulated genes (*APOA1, SOCS2, CCL21, ODC1, CYP2J2, DGKG, FGF1*, and *INPP4B*) and 10 down-regulated genes (*ETNPPL, SGPP2, PLA2G2A, PLIN2, ALOX5AP, G0S2, LPCAT3, EBP, PTDSS1*, and *IMPA2*) (Figure 2D).

### Gene Ontology Enrichment Analysis of the Differentially Expressed Lipid Metabolism-Related Genes

**FIGURE 3:**
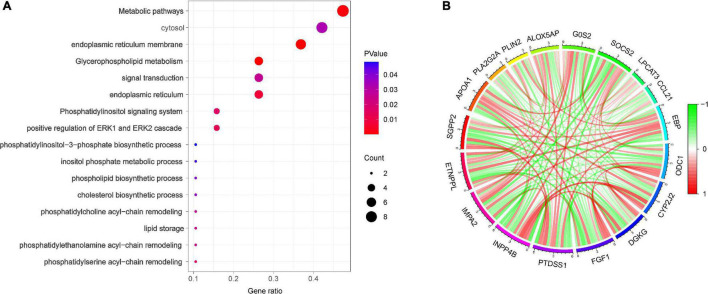
Functional enrichment analysis **(A)** Bubble plot of enriched GO terms. **(B)** Spearman correlation analysis of the 18 DELs.

Gene ontology analysis showed that the biological process of DELs focused primarily on positive regulation of ERK1 and ERK2 cascades, phosphatidylserine acyl-chain remodeling, phosphatidylethanolamine acyl-chain remodeling, lipid storage, and signal transduction. The main cellular components included endoplasmic reticulum (ER) membrane, ER, and cytosol (Figure 3A).

To identify the most important regulatory molecule in the 18 DELs, we analyzed correlations between the DELs (Figure 3B). The results showed that INPP4b was positively correlated with CYP2J2 and DGKG, but negatively correlated with PTDSS1 and IMPA2.

### Validation of the Differentially Expressed Lipid Metabolism-Related Genes Associated With Dilated Cardiomyopathy

**FIGURE 4:**
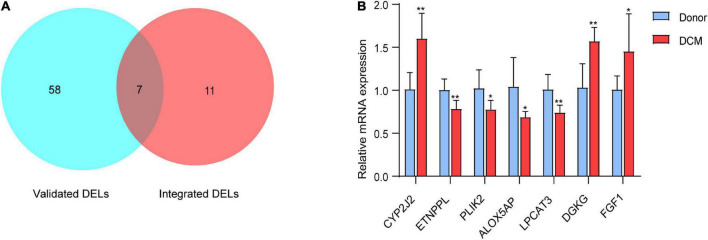
Identify 7 differentially expressed lipid metabolism-related genes. **(A)** Venn diagram of differentially expressed lipid metabolism-related genes based on validation sets (EGAS00001003263) and the integrated microarray dataset. **(B)** RT-qPCR analysis for mRNA levels of the in the mRNA expression of the 7 DELs in dilated hearts (red bars) vs. Donor hearts (blue bars). The values from the Donors were set to 1. Data are presented as mean ± SEM. *P < 0.05, **P < 0.01 vs. Donors.

Next, we used the external validation sets (EGAS00001003263) to further verify the accuracy of the transcriptome data. A total of seven DELs, including *CYP2J2, FGF1, ETNPPL, PLIN2, LPCAT3, ALOX5AP* and *DGKG*, were found to be differentially expressed in both EGAS00001003263 and the integrated dataset (Figure 4A). The seven DELs were further validated by RT-PCR in five healthy individuals and five DCM patients. As shown in Figure 4B, compared with the donor group, the expressions of *CYP2J2, FGF1*, and *DGKG* were up-regulated, which was consistent with the results of bioinformatics analysis. In addition, *ETNPPL, PLIN2, ALOX5AP*, and *LPCAT3* were down-regulated in human DCM heart samples.

### Correlation Analysis Between Differentially Expressed Lipid Metabolism-Related Genes and Clinical Factors

**FIGURE 5:**
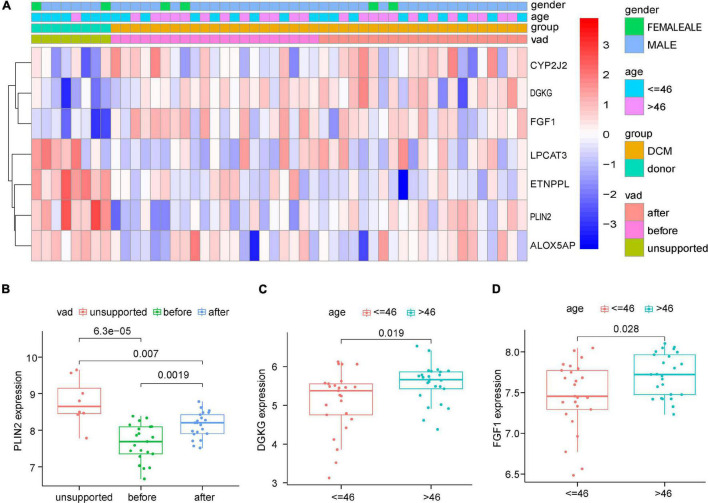
Correlation analysis based on Clinical informations and expression profiles of the 7 DELs in GSE21610. **(A)** Heatmap of correlation analysis. **(B)** The expression levels of PLIN2 were significantly upregulated after VAD supported. **(C,D)** The expression levels of DGKG and FGF1 were significantly upregulated in elder group (>46 years).

We then chose the GSE21610 dataset, which provides more comprehensive clinical information, to investigate the relationships between the expression levels of the seven DELs and clinical factors (Figure 5A). The box plots showed that compared with the expression patterns before ventricular assisted device (VAD) support, the expression of *PLIN2* increased after VAD support in DCM (Figure 5B). Age plays an important role in lipid metabolism dysfunction. *DGKG* and *FGF1* were up-regulated at older age (≥46 years), indicating a positive correlation between age and the expression levels of *DGKG* and *FGF1* (DGKG: *p* = 0.019; FGF1: *p* = 0.028) (Figures 5C,D). However, there were no significant differences in the clinical feature of gender and expression pattern.

### Six-Gene-Based Logistic Regression Model

**FIGURE 6:**
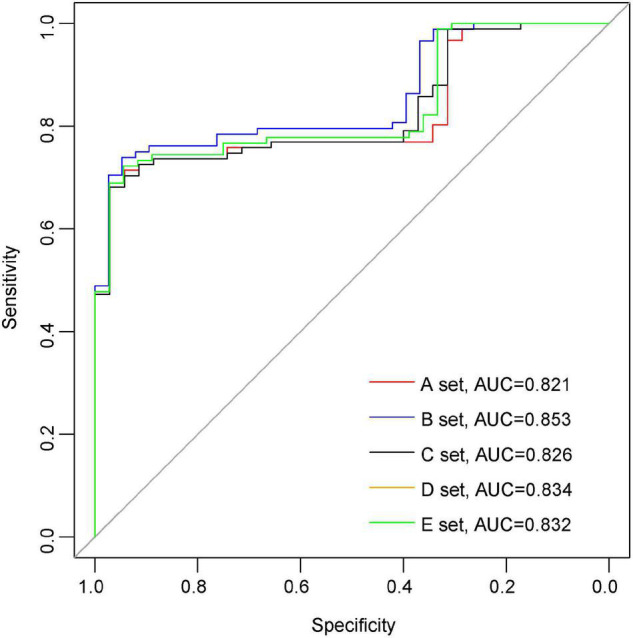
Establishment of logistic regression model. The Receiver operating characteristic (ROC) curve for predicting overall outcomes by logistic regression model.

In order to further elucidate the clinical significance of the seven DELs, we applied the Lasso regression method using “glmnet” R package to the seven DELs. The samples were randomly divided into a training set and a testing set, where the training set accounted for 70% of the samples, and this step was repeated five times to reduce the error and enhance the sensitivity of the models. Finally, six genes were retained, including *CYP2J2*, *FGF1*, *ETNPPL*, *PLIN2*, *LPCAT3*, and *DGKG*. The area under the ROC curve (AUC) was used to assess the accuracy of the models. The AUC values of the five models in mRNA dataset GSE36961 were 0.821, 0.853, 0.826, 0.834, and 0.832, suggesting good explanatory power of the model (Figure 6). Taken together, the logistic regression model established based on the six DELs could effectively identify sample type (DCM/healthy control), and *CYP2J2*, *FGF1*, *ETNPPL*, *PLIN2*, *LPCAT3*, and *DGKG* were potential targets for DCM study.

## Discussion

Previous studies have used GEO databases to identify differentially expressed genes by microarray analysis, which is helpful for early diagnosis and reduction of misdiagnosis rate. To date, the application of microarray analysis has revealed a large number of biological processes associated with DCM (15, 16). In this study, we identified 18 DEGs related to lipid metabolism through bioinformatics, and finally obtained seven DEGs related to lipid metabolism through external dataset verification. RT-PCR was used to further verify the expression of these seven genes. Finally, a logistic model was established and six of these genes were confirmed to be of diagnostic significance.

Dilated cardiomyopathy is a widespread cardiac phenotype that can be caused by various myocardial injuries (17). The high incidence and mortality of DCM underscores the clinical importance of exploring DCM. To explore the underlying mechanism of DCM, we firstly performed GSVA analysis and found fatty acid metabolism was a relatively significant feature in DCM. In recent years, a decrease in fatty acid oxidation has been observed in DCM (18, 19). Our results also confirmed the close relationship between DCM and lipid metabolism from the perspective of bioinformatics. However, lipid metabolism disorders in DCM remain poorly understood. This aroused our great interest. Considering individual differences and technical limitations, we integrated three gene expression profile datasets to obtain a larger sample size to improve the accuracy of the conclusions. We downloaded a list of genes associated with lipid metabolism and validated their expression in the integrated dataset. In our work, a total of 19 DELs were identified, including eight up-regulated genes and 11 down-regulated genes. The up-regulated genes include Apolipoprotein A1 (APOA1), a main protein in high-density lipoprotein (HDL) (20), but many of the other genes related to lipid metabolism have rarely been reported. Another study also found that APOA1 was significantly differentially expressed between non-heart failure and heart failure patients, and could be a candidate blood protein biomarker of heart failure (21). The present study identified numerous DELs associated with DCM, which could be potential targets for subsequent research. Functional annotation of DELs was performed to understand the processes and pathways in which they participate. GO analysis revealed that metabolic pathways, glycerophospholipid metabolism and signal transduction were major biological processes in which the DELs were involved. Then, we validated the expression levels of DELs by both bioinformatics analysis in EGAS00001003263 dataset and RT-PCR analysis in human DCM and healthy donor heart samples. Combined with the results of comprehensive bioinformatics analysis and gene expression measurements, this study identified potential key genes and provided high-value targets for subsequent therapeutic studies in DCM.

There are individual differences in DCM patients, such as age, gender, and whether or not a VAD is implanted. Previous studies have shown that gene expression levels in heart failure patients correlate with age. Fibroblasts from DCM patients aged > 6 years show profound alterations in gene expression patterns, mainly in the form of enrichment of genes encoding fibrillar collagen, regulation of proteoglycans, conversion of thrombochondroitin isoforms, and features of fibroblast activation (22). Familial DCM is usually chromosomally dominant, but familial DCM predominantly affects males, with a high reported male-to-female ratio of 1.5:1 (23). Herman et al. reported various mutations in TTN (the gene encoding the sarcoma protein titin) in 312 patients with idiopathic DCM and found that men carrying TTN mutations had an earlier onset of adverse events than women (24). X-linked cardiomyopathy, caused by a defect in the dystrophin gene, is an inherited disease. In a large cohort of consecutive male DCM patients, the article noted that 6.5% of patients were found to have dystrophin gene defects (25). Altered gene expression, particularly in the extracellular matrix (ECM), can occur after left ventricular assist device (LVAD) implantation. Patients recovered from LVAD implantation with reduced expression levels of pro-fibrotic genes (26). Matrix metalloproteinases (MMPs) and the tissue inhibitors of metalloproteinases (TIMPs) were generally higher in the LVAD group (27). These suggest that lipid metabolism-related gene expression levels may also be influenced by these individual differences during the occurrence of DCM. The present study therefore next analyses whether these common individual differences such as age, gender and whether VAD is implanted may be associated with the expression levels of lipid metabolism-related genes. The results showed that the expression levels of DGKG and FGF1 were positively correlated with age, while only the expression level of PLIN2 was correlated with the presence or absence of VAD implantation (Figures 5B–D).

Since there is still lack of evidence whether these lipid metabolism-related genes contribute to the diagnosis of dilated cardiomyopathy, a logistic regression model was constructed to distinguish patients with or without DCM based on the six key genes. The six genes are *CYP2J2, FGF1, ETNPPL, PLIN2, LPCAT3*, and *DGKG.* CYP2J2, a member of the cytochrome P450 family of enzymes in the heart, is involved in the metabolism of fatty acids (28). Over-expression of CYP2J2 in endothelium was found to improve cardiac function by increasing angiogenesis in MI-induced heart failure (29), whereas over-expression of CYP2J2 by tail vein injection of rAAV9-CYP2J2 was found to attenuate ethanol-induced myocardial dysfunction (30). Emerging evidence suggests that fibroblast growth factor-1 (FGF1), which induces adipocyte precursor differentiation through activation of the FGF-receptor-1 (31), has a beneficial effect in heart diseases. FGF1 is involved in the regulation of cardiac myocyte regeneration. Nanoparticle-mediated delivery of FGF1 was found to enhance the engraftment and regenerative potency of transplanted human cardiomyocyte patches (hCMPs) after myocardial infarction in mice (32), while FGF1/p38 MAP kinase inhibitor treatment after acute myocardial injury in 8 to 10-week-old rats was found to induce cardiomyocyte mitosis (33). FGF1 coacervate inhibited ventricular dilation and preserved cardiac contractibility better than free FGF1 and saline control in a mouse model of acute myocardial infarction (34). Ethanolamine-phosphate lyase (ETNPPL/AGXT2L1), a lipid metabolizing enzyme, is involved in the catabolism of ethanolamine phosphate in membrane synthesis (35). However, the role of ETNPPL in cardiac disease has not been reported. Perilipin 2 (Plin2) is a lipid droplet (LD)-associated protein that is involved in intracellular LD formation, stabilization and trafficking events. Cardiac over-expression of Plin2 leads to massive myocardial steatosis, suggesting that Plin2 stabilizes LD (36). Transgenic (Tg) mice with hearts specifically over-expressing PLIN2 developed cardiac steatosis, and ultrasound showed a significantly longer RR interval than WT mice (37). In PLIN2-/- mice, PLIN2 deficiency significantly increased triglyceride accumulation in cardiomyocytes (38). Lysophosphatidylcholine acyltransferase 3 (Lpcat3) is a key factor in lipoprotein production and control metabolic pathways, lacking Lpcat3 in the liver reduced plasma TGs, hepatosteatosis, and secreted lipid-poor very low-density lipoprotein (VLDL) without arachidonoyl PLs (39, 40). The impact of LPCAT3 on heart disease remains unknown. Diacylglycerol kinase (DGK) belongs to a class of enzymes that catalyze the conversion of diglycerides to phosphatidic acid, and is considered to be a key regulator of cellular signaling pathways (41). Compared with other DGK isozymes, diacylglycerol kinase gamma (DGKG) showed a strong binding activity for phosphatidic acid (PA) (42). Given the limited understanding of these DEGs in DCM, their specific biological functions and molecular regulatory mechanisms need to be confirmed by further studies. As previous analyses showed that age was strongly correlated with the expression levels of DGKG and FGF1, we randomized the groups by age and sex, thus excluding the effect of confounding factors such as age on the predictive accuracy of the model. However, further research methods are needed to increase the reliability of the model.

This study had several limitations. First, the sample size of the GEO database is small. Second, due to the shortcomings of the study itself, the conclusions need to be validated by basic research and clinical trials. In addition, the study only verified the mRNA level by RT-PCR, but not the protein level.

## Data Availability Statement

The datasets presented in this study can be found in online repositories. The names of the repository/repositories and accession number(s) can be found in the article/Supplementary Material.

## Ethics Statement

The studies involving human participants were reviewed and approved by the Review Committee of the People’s Hospital of Wuhan University. Written informed consent to participate in this study was provided by the participants’ legal guardian/next of kin. Written informed consent was obtained from the individual(s), and minor(s)’ legal guardian/next of kin, for the publication of any potentially identifiable images or data included in this article.

## Author Contributions

MX and Y-YG contributed equally to this work. All authors listed have made a substantial, direct, and intellectual contribution to the work, and approved it for publication.

## Conflict of Interest

The authors declare that the research was conducted in the absence of any commercial or financial relationships that could be construed as a potential conflict of interest.

## Publisher’s Note

All claims expressed in this article are solely those of the authors and do not necessarily represent those of their affiliated organizations, or those of the publisher, the editors and the reviewers. Any product that may be evaluated in this article, or claim that may be made by its manufacturer, is not guaranteed or endorsed by the publisher.
